# Anticipating the Prevalence of Avian Influenza Subtypes H9 and H5 in Live-Bird Markets

**DOI:** 10.1371/journal.pone.0056157

**Published:** 2013-02-07

**Authors:** Kim M. Pepin, Jia Wang, Colleen T. Webb, Jennifer A. Hoeting, Mary Poss, Peter J. Hudson, Wenshan Hong, Huachen Zhu, Yi Guan, Steven Riley

**Affiliations:** 1 Fogarty International Center, National Institutes of Health, Bethesda, Maryland, United States of America; 2 Department of Biology, Colorado State University, Fort Collins, Colorado, United States of America; 3 International Institution of Infection and Immunity, Shantou University Medical College, Shantou, People’s Republic of China; 4 State Key Laboratory of Emerging Infectious Diseases, The University of Hong Kong, Hong Kong SAR, People’s Republic of China; 5 Department of Statistics, Colorado State University, Fort Collins, Colorado, United States of America; 6 Department of Biology, Center for Infectious Disease Dynamics, Pennsylvania State University, University Park, Pennsylvania, United States of America; 7 Department of Infectious Disease Epidemiology, School of Public Health, Imperial College London, London, United Kingdom; University of Oxford, Viet Nam

## Abstract

An ability to forecast the prevalence of specific subtypes of avian influenza viruses (AIV) in live-bird markets would facilitate greatly the implementation of preventative measures designed to minimize poultry losses and human exposure. The minimum requirement for developing predictive quantitative tools is surveillance data of AIV prevalence sampled frequently over several years. Recently, a 4-year time series of monthly sampling of hemagglutinin subtypes 1–13 in ducks, chickens and quail in live-bird markets in southern China has become available. We used these data to investigate whether a simple statistical model, based solely on historical data (variables such as the number of positive samples in host X of subtype Y time *t* months ago), could accurately predict prevalence of H5 and H9 subtypes in chickens. We also examined the role of ducks and quail in predicting prevalence in chickens within the market setting because between-species transmission is thought to occur within markets but has not been measured. Our best statistical models performed remarkably well at predicting future prevalence (pseudo-R^2^ = 0.57 for H9 and 0.49 for H5), especially considering the multi-host, multi-subtype nature of AIVs. We did not find prevalence of H5/H9 in ducks or quail to be predictors of prevalence in chickens within the Chinese markets. Our results suggest surveillance protocols that could enable more accurate and timely predictive statistical models. We also discuss which data should be collected to allow the development of mechanistic models.

## Introduction

H5 and H9 subtypes of avian influenza viruses (AIV) are two of the three avian subtypes (H7 is the third) known to cause infection in humans [1], [2]. H5 and H9 continue to be isolated from live-bird markets in multiple countries [3], [4], [5] and thus pose an ongoing public health threat as potential pandemic strains. Quantitative tools for anticipating prevalence patterns of these subtypes in markets are needed to improve prevention and response plans in a cost-effective manner. Forecasting the future is challenging in any complex biological system, but is particularly difficult for AIVs in live-bird markets because of: the intricate host population ecology, the rarity of some subtypes that can cause infection in humans (i.e., H5 and H7), and the lack of comprehensive longitudinal prevalence data for multiple subtypes. Recently, we reported 6 years of monthly prevalence data for Hemagglutinin (H) subtypes 1–13 [4] in multiple host species in live-bird markets in Shantou, China. To our knowledge, this is the most comprehensive longitudinal time series of AIV prevalence in a domestic poultry setting. The study found striking patterns of host specificity and co-infection bias between subtypes, highlighting that host species composition and the prevalence of multiple subtypes are key in determining subtype-specific prevalence patterns in southern Chinese markets.

The three subtypes of avian influenza that have occurred naturally in humans thus far are H5, H7 and H9 [6], [7], [8], [9], . Highly pathogenic avian influenza H5N1 causes acute disease in most human cases, with death in >60%, whereas H7 tends to cause conjunctivitis and H9 tends to cause mild influenza-like-illness [1]. However, although cases of H5N1 are reported most frequently, growing evidence shows that H9 may occur more often in humans in China than H5 or H7 [15], [16], [17]. Moreover, the host range of H9 overlaps with H5 [4], which presents an opportunity for H9 to acquire genetic material from strains that are virulent in humans. In live-bird markets in southern China, H9 is the most prevalent subtype while H5 is relatively rare and H7 is very rare [4]. Thus, in southern China, H9 and H5 present the largest risk of spill-over infections to humans. While many influenza A subtypes have a strong host preference for ducks, H9 is well-adapted to chicken and quail, and H5 is adapted to all three of these dominant host species [4].

The main goal of the analyses described here was to investigate whether a regression model, with biologically interpretable parameters, could be developed from surveillance data as an easy-to-use tool for anticipating the prevalence of H9 or H5 in Chinese live-bird markets. As a case study, we focused on prevalence in chickens because this poultry species is a major staple with relatively high AIV prevalence. In addition, we used the statistical framework to investigate whether AIV prevalence in other host species was associated with prevalence levels in chickens. Lastly, we conducted model selection to identify which surveillance data were most crucial for predicting prevalence of H9 and H5. We discuss which missing data would likely improve model accuracy.

## Materials and Methods

### Data Collection

Routine sampling of *Anas platyrhynchos* (duck, domestic and wild), *Coturnix japonica* (Japanese quail) and *Gallus gallus* (domestic chicken and silkie chicken) in nine live bird markets was conducted at 2–4 week intervals in the city of Shantou, China from November 2002 through October 2006. The data were part of a larger surveillance effort that included samples from other host species and over a longer period (2000–2006) [4], but here we restrict the data to the most intensely sampled species during the time frame that sampling methods were consistent.

Cloacal and tracheal swabs were collected from each bird. Birds were counted as positive if virus was isolated from at least one of the two samples. Virus was isolated using embryonated chicken eggs and AIV subtypes H1-13 and Avian Paramyxovirus-type-1 (APMV-1) were identified using monospecific antisera in hemagglutination inhibition (HI) tests [4]. For more details of the data collection methods see [4].

### Data Organization

Positive counts were aggregated at a monthly scale and transformed to counts per 100 birds ([count/sample size] x 100), which is close to the mean sample sizes (see below). Data from H2, H7, H8, H10, H12 and H13 were discarded since these subtypes were very rare. Our previous work identified strong host preferences between subtypes, and different sample sizes for each host species were collected, thus it was important to model prevalence within individual host species [4]. We focused on modeling the prevalence of H5 and H9 since they are the two subtypes that infect chickens most frequently and are of public health concern. Potential covariates for each model included prevalence of: H1, H3, H4, H5, H6, H9, and H11 (with the exclusion of the focal subtype) in each of the 3 hosts. Since environmental transmission through water sources is an important mode of AIV transmission [18], [19] and weather can affect virus stability in water [20], [21] and has been associated with broad patterns of virus prevalence [22], we also included 12 local weather variables: mean temperature, maximum temperature, minimum temperature, humidity, precipitation, visibility, wind speed, and maximum wind speed (http://www.tutiempo.net). We considered temperature because it is known to affect virus stability and thus could affect rates of environmental transmission [20], [23]. We considered precipitation and humidity because they have measurable effects on both direct and indirect transmission of influenza A [24], [25]. Although there are no data describing effects of visibility and wind speed on transmission, we considered these variables because we hypothesized that they could affect either behavior of merchants or airborne transmission (as in [26]).

Covariates were considered within the same time step and at a lag of one month because, by observation, peaks of incidence are of that approximate duration. Hence, if weather variables contribute to prevalence peaks, we might expect high prevalence to occur ∼one month after a rise or dip in weather values. Furthermore, since the infectious period and transition times through the market are very short (∼ 5–10 days and 2–3 days, respectively), we did not expect prevalence of other subtypes to affect prevalence of H5 or H9 at more than one time step in the past (i.e., one month). All covariates were normalized by taking the difference from each point to the mean of all points and then dividing the result by the standard deviation. Because the infectious period is relatively short (usually <1 week [27], [28]), covariates from within the same monthly time step should be most relevant. The first 36 months of data were used for model selection while the last 12 months were reserved for assessing forecasts from the model with the best set of covariates. Bird species sample sizes (mean ±2 standard errors) for these two time periods were: 154.8±14.2 and 117.9±14.4 for ducks, 89.3±11.2 and 84.0±16.4 for chicken, and 40.6±5.0 and 40.1±4.9 for quail. Thus, fewer duck samples were collected during the last 12 months relative to the first 36 months but this should not affect results because both mean sample sizes are quite high and data were expressed as a function of host species sample size.

### Model Structure

We used generalized linear modeling. H9 data were modeled with a negative binomial (NB) error structure (log link; ‘glm.nb’ function in the ‘MASS’ package in R 2.15.1 [29]) and H5 data with a zero-inflated negative binomial model (ZINB; log link for the negative binomial component and logit link for the zero-inflated component; ‘zeroinfl’ function in the ‘pscl’ package in R 2.15.1 [30]). We chose a log link for the negative binomial component since the covariates showed no clear relationship to the response (neither linear nor multiplicative) and the models would not converge using the identity link. Residual analyses showed that these model structures were the most appropriate when compared to Poisson, quasi-Poisson, zero-inflated Poisson and Poisson autoregressive models. Residuals for the chosen models showed no significant autocorrelation over time.

### Model Selection

First, we tested whether the prevalence of H9 and H5 in chickens in retail markets is associated with the prevalence of these subtypes in the other host species (this model is referred to as “DK+QA” since it includes parameters for subtype prevalence in ducks and quail), by constructing models with only these data (H9 or H5 in ducks and quail, separately) as covariates. Second, in order to examine forecast ability and to identify which variables were important for predicting the prevalence of H9 and H5, we conducted model selection using prevalence data from the other subtypes in each of the 3 hosts and weather data. We included data from the same time step as well as data from 1 month in the past. Due to the large number of possible models, we performed a preliminary step and fit all single variable models and selected variables which improved model AIC by at least 2 points over the intercept-only model. From this subset of variables, we fit all possible combinations and selected the top model (referred to as “Best”) by AIC.

### Model Evaluation

We assessed the appropriateness of models by probability plots and autocorrelation function plots of residuals, and Vuong tests [31]. We evaluated fits by Cragg and Uhler’s pseudo-Rsquared [32], which is a measure of how much better the full model performs relative to the intercept-only model on a scale of 0 to 1, where 1 means the model fits the data perfectly, and 0 means the model does no better than the intercept-only regression model. We compared accuracy of in-sample model predictions to model forecasts by mean squared prediction errors (MSPE) and by normalized mean squared prediction errors (NMSPE) [33]. The former method emphasizes deviations from large peaks in the data [33] while the latter is an overall empirical-to-model variability rate [34]. We forecasted data using 3 different methods: 1) forecasting 1-year of monthly data from a model fit to the first 3 years (“Full”), 2) forecasting one step at a time by iterative fitting to all prior months and prediction of one month in the future (Step-by-step A), and 3) forecasting one step at a time using a sliding window for fitting - fitting to the previous 36 months and predicting the next month in the future (Step-by-step B).

## Results

**Figure 1:**
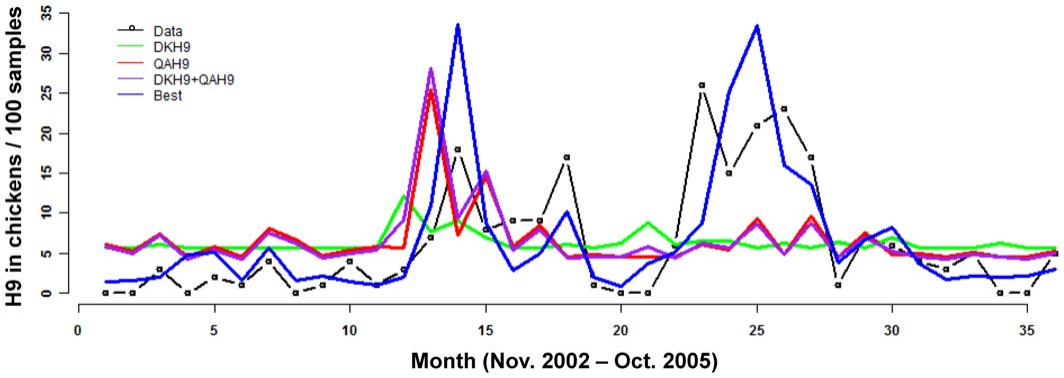
Model fits for H9. Data at the prevalence of H9 per 100 chickens sampled. Data were modeled by negative binomial regression with a log link. “Best” is the set of covariates that were selected by AIC: allH4, allH6, QAH6, QAH9, allH5_t-1_, where “all” is the prevalence of subtype HX in all 3 host species (CK+DK+QA), “QA” is for prevalence in only quail, “DK” is for prevalence in only duck, and t-1 is the prevalence in the previous month.

First, we fit models of H9 in chickens using H9 prevalence in ducks and quail as covariates to evaluate whether these other host species of H9 are associated with H9 prevalence in chickens. H9 in ducks and quail were not correlated with H9 in chickens when considered on their own (Table 1, Figure 1). A model with these covariates at a one-month lag performed even more poorly than when they were considered in the same month (data not shown). This suggests that transmission of H9 from these other hosts to chickens is not significant within the retail market context. The best model included H4 and H6 in all 3 host species during the same month, H5 in all host species during the previous month, and H6 and H9 in quail during the same month. For all covariates except for H6 (in all 3 host species), H9 prevalence increased with increasing values of the covariates (Table 2). H6 in quail and H5 in all hosts in the previous month were highly significant (Table 2), while the other covariates were marginally not significant.

**Table 1:** Models of H9 that include H9 data in other hosts compared to the “best” model selected.

Model	AIC	1pseudo-R^2^	2MSPE	3NMSPE
**Intercept**	211.9	0	52.0	NA
**DKH9**	213.4	0.01	52.2	30.8
**QAH9**	211.5	0.06	56.3	3.8
**DKH9+QAH9**	213.2	0.07	59.2	3.2
4 **Best**	191.8	0.57	30.0	0.4

1 Cragg & Uhler’s method: (1-(L_0_/L_m_)^2/N^ )/1-L_0_^2/N^; L = likelihood; 0 = intercept-only model; m = full model; N = number of data points.

2 Mean Squared Prediction Error: sum(y-m)^2^/N; smaller values indicate better fits; y = observed data; m = mean of predicted data; N = number of points predicted.

3 Normalized Mean Squared Prediction Error: sum((y-m)/s)^2^/N; smaller values indicate better fits; y = observed data; m = mean of predicted data; s = standard deviation of predicted data; N = number of points predicted.

4 Best model covariates: H4 prevalence in all hosts, H6 prevalence in all hosts, H6 prevalence in quail, H9 prevalence in quail, H5 prevalence in all hosts one month in the past.

Covariates in other models: DKH9 = H9 prevalence in ducks, QAH9 = H9 prevalence in quail, DKH9+QAH9 = sum of DKH9 and QAH9.

**Table 2:** Parameter estimates for the best model of H9 in chickens.

Covariate	Estimate	SE	P
**Intercept**	1.24	0.15	<0.0001
**H4 prevalence in all hosts**	0.28	0.15	0.059
**H6 prevalence in all hosts**	−0.56	0.29	0.058
**H6 prevalence in quail**	1.05	0.29	0.0003
**H9 prevalence in quail**	0.24	0.13	0.064
**H5 prevalence in all hosts** **1 month in the past**	0.41	0.13	0.0021

Model covariates: allH4 = H4 prevalence in all hosts, allH6 = H6 prevalence in all hosts, QAH6 = H6 prevalence in quail, QAH9 = H9 prevalence in quail, allH5_t-1_ = H5 prevalence in all hosts one month in the past.

**Figure 2:**
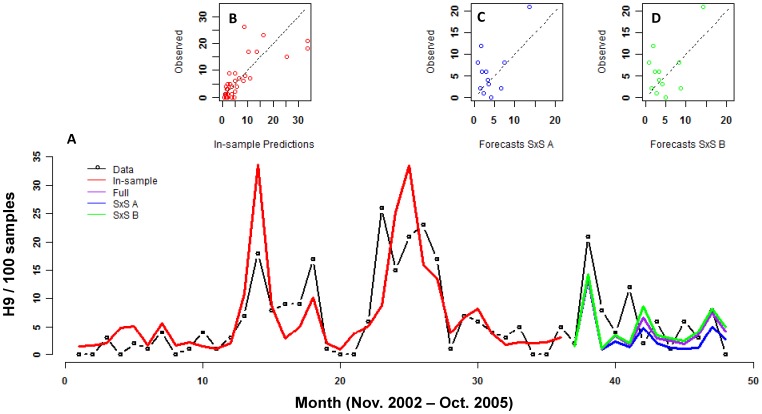
Forecasts with the best model for H9. The model was fit (red) on the first 3 years of data (black). Forecasts are shown for the fourth year of data using 3 methods: 1) Forecasting the full 12 months of data (blue), 2) Iterative fitting and forecasting where additional data were included at each step (SxS A, purple), and 3) Iterative fitting and forecasting using a sliding window where model parameters were always estimated from 36 months of data (SxS B, green). B-D show an alternative way of viewing the fits. B shows the fit of the model and C and D show the fit of the forecasted points using the two best methods (SxS A (C) and SxS B (D)).

The best model of H9 prevalence fit the data quite well both quantitatively (pseudo-R^2^ = 0.57; large reduction in MSPE relative to the intercept-only model; Table 1) and qualitatively (captured the timing of major peaks and periods of low prevalence, Figure 1) considering the multi-host, multi-subtype nature of AIV ecology. The model also performed well at predicting future data (Figure 2), especially when an iterative fit and out-of-sample prediction (step-by-step B) approach was used (Table 3 and Figure 2). On average, the model produced smaller deviations from large peaks in the predicted data relative to the fitted data (see MSPE in Table 3), but this was because the predicted data had smaller peaks overall (Figure 2). The NMSPE in Table 3 shows that the prediction errors are not much larger than errors from the fitted model. Also, the timing of the largest peak was captured and the model did not predict large peaks where none occurred.

**Table 3:** Evaluation of best model of H9 in chickens.

Method	MSPE	NMSPE
**In-sample data**	30.0	0.4
**12-month forecast**	24.5	1.9
**Step-by-step A**	23.1	1.8
**Step-by-step B**	24.1	1.6

In-sample data are for the fitted model. Other methods are described in Figure 2. Note that MSPE emphasizes deviations from larger peaks. The in-sample data show poorer performance relative to the forecasts since the first 3 years of data contained several much larger peaks than the last year of data.

Mean Squared Prediction Error (MSPE): sum(y-m)^2^/N; smaller values indicate better fits; y = observed data; m = mean of predicted data; N = number of points predicted.

Normalized Mean Squared Prediction Error (NMSPE): sum((y-m)/s)^2^/N; smaller values indicate better fits; y = observed data; m = mean of predicted data; s = standard deviation of predicted data; N = number of points predicted.

**Figure 3:**
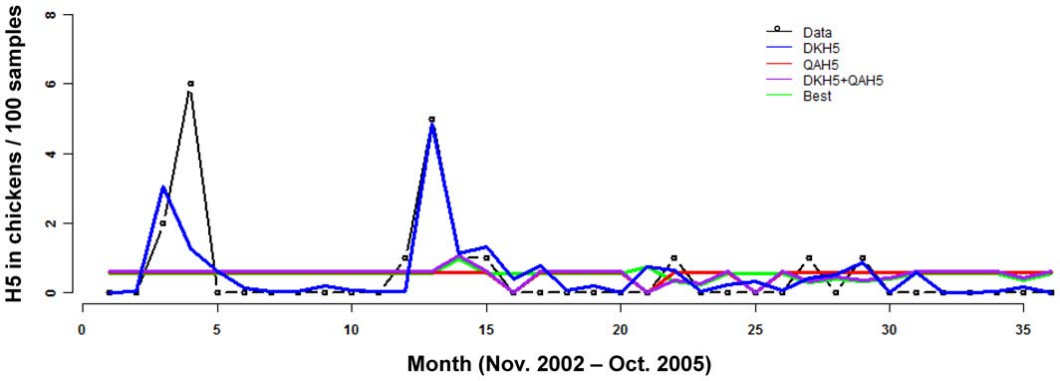
Model fits for H5. Data are the prevalence of H5 per 100 chickens sampled. Data were modeled by zero-inflated negative binomial regression with a log link on the abundance component. “Best” is the set of covariates that were selected by AIC: maximum wind speed and DKH9_t-1_, where “DK” is for prevalence in ducks, and t-1 is the prevalence in the previous month.

**Figure 4:**
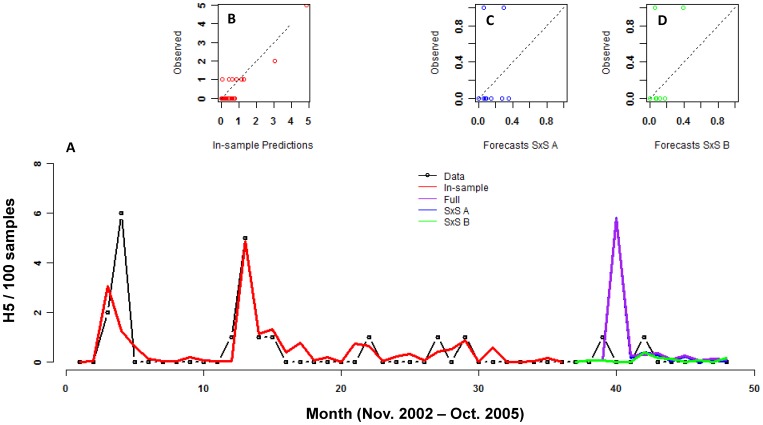
Forecasts with the best model for H5. The model was fit (red) on the first 3 years of data (black). Forecasts are shown for the fourth year of data using 3 methods: 1) Forecasting the full 12 months of data (blue), 2) Iterative fitting and forecasting where additional data were included at each step (SxS A, purple), and 3) Iterative fitting and forecasting using a sliding window where model parameters were always estimated from 36 months of data (SxS B, green). B-D show an alternative way of viewing the fits. B shows the fit of the model and C and D show the fit of the forecasted points using the two best methods (SxS A (C) and SxS B (D)).

Modeling H5 data was more challenging because H5 is rare relative to H9 and its rarity increased in the last year of the time series, the section used for out-of-sample prediction. Similar to H9, H5 in ducks and quail were not correlated with H5 in chickens (Table 4, Figure 3). The best model included only two covariates: H9 in ducks in the previous month and maximum wind speed. In fact, very few single-variable models fit the data even moderately well and these only included H9 prevalence and weather variables (data not shown). The best model fit the data well both quantitatively (pseudo-R^2^ = 0.49, Table 4) and qualitatively (the timing of major peaks is captured). The parameters for the negative binomial component of the zero-inflated negative binomial model indicated that as maximum windspeed and prevalence of H9 in ducks increased, prevalence of H5 in chickens also increased (P<0.017 and P<0.022, respectively; Table 5). The role of these parameters in causing excess zeros was less clear because the standard errors on these estimates were very large (Table 5, the binomial component). The first two methods of out-of-sample prediction (Full and Step-by-step A) performed poorly in anticipating future H5 prevalence (i.e., they predicted large outbreaks that did not occur) while the step-by-step method B did quite well considering the data challenges (i.e., it predicted no peaks and none occurred; Figure 4, Table 6). The large values for NMSPE (which indicate lack of fit) are due to the low absolute values in the last year of observed data as well as the small standard deviation in the predicted time series. For Method step-by-step B, the MSPE is the better statistic for evaluating the out-of-sample predictions.

**Table 4:** Models of H5 that include H5 data in other hosts compared to the “best” model selected.

Model	AIC	1pseudo-R	2MSPE	3NMSPE
**Intercept**	71.8	0	1.7	NA
**DKH5**	71.4	0.14	1.7	111.1
**QAH5**	74.1	0.06	1.7	64.1
**DKH5+QAH5**	73.1	0.20	1.7	39.5
4 **Best**	60.9	0.49	0.8	0.8

Column statistics are by the same methods as described in Table 1.

1 Cragg & Uhler’s method: (1-(L_0_/L_m_)^2/N^ )/1-L_0_^2/N^; L = likelihood; 0 = intercept-only model; m = full model; N = number of data points.

2 Mean Squared Prediction Error: sum(y-m)^2^/N; smaller values indicate better fits; y = observed data; m = mean of predicted data; N = number of points predicted.

3 Normalized Mean Squared Prediction Error: sum((y-m)/s)^2^/N; smaller values indicate better fits; y = observed data; m = mean of predicted data; s = standard deviation of predicted data; N = number of points predicted.

4 Best model covariates: Maximum windspeed, H9 prevalence in ducks one month in the past.

Covariates in other models: DKH5 = H5 prevalence in ducks, QAH5 = H5 prevalence in quail, DKH5+QAH5 = sum of DKH5 and QAH5.

**Table 5:** Parameter estimates for the best model of H5 in chickens.

Component	Covariate	Estimate	SE	P
**Negative Binomial**				
	**Intercept**	−0.96	0.58	0.099
	**Max Windspeed**	1.04	0.43	0.017
	**Prevalence of H9 in ducks on month in the past**	0.47	0.20	0.022
**Binomial**				
	**Intercept**	−2.81	8.63	0.74
	**Max Windspeed**	−0.60	0.94	0.53
	**Prevalence of H9 in ducks on month in the past**	−8.81	17.08	0.61

1 The zero-inflated negative binomial model is a mixture of two separate data generation processes (i.e., model “components”): one to describe zeros (binomial) and the other to describe counts from a negative binomial model.

DKH9_t-1_ = H9 prevalence in ducks one month in the past.

**Table 6:** Evaluation of best model of H5 in chickens.

Method	MSPE	NMSPE
**In-sample data**	0.8	0.8
**12-month forecast**	2.9	1.1
**Step-by-step A**	2.9	1.1
**Step-by-step B**	0.1	8.0

In-sample data are for the fitted model. Other methods are described in Figure 2. Column statistics are by the same methods as described in Table 3.

Mean Squared Prediction Error (MSPE): sum(y-m)^2^/N; smaller values indicate better fits; y = observed data; m = mean of predicted data; N = number of points predicted.

Normalized Mean Squared Prediction Error (NMSPE): sum((y-m)/s)^2^/N; smaller values indicate better fits; y = observed data; m = mean of predicted data; s = standard deviation of predicted data; N = number of points predicted.

## Discussion

Anticipating the prevalence of specific subtypes of AIV in domestic poultry settings is critical for planning and implementing cost-effective public health preventions. To date, it has not been possible to forecast the prevalence of any AIV subtype in poultry because the appropriate data have not been available and the ecology and population dynamics of AIV are complex. Here, we evaluated the possibility that surveillance data that were collected for purposes other than our analyses could be used both to gain information on factors that may influence the dynamics of AIV within live-bird markets in southern China and to create a tool for predicting prevalence. Our two most important findings were that: prevalence of H9 and H5 in chickens was uncorrelated with prevalence of these subtypes in ducks or quail *within the market environment*, and models that produce reasonably good predictions could be made so long as data from other subtypes and host species were included.

One striking difference between the best models of H9 and H5 was that H5 dynamics were mainly associated with environmental variables (except for the influence of H9) whereas H9 was unaffected by weather and mainly associated with the dynamics of other subtypes. Our previous study [4] found that H5 was the only subtype that did not show host specificity of co-infection patterns. These two observations together suggest that the dynamics of this subtype are inherently different from other AIVs. Interestingly, there appeared to be some positive association between these two subtypes - that is H9 increased when H5 increased in all host species in the previous month, and H5 increased when H9 increased in ducks in the previous month, suggesting that when an outbreak of one of these subtypes is anticipated, preparation for the other should be considered.

A second difference between H9 and H5 was in the relative performance of the alternate methods of out-of-sample prediction. The step-by-step method was best for both H5 and H9, but it was crucial to use the step-by-step sliding window approach for H5. This was because the time series shows an obvious change in prevalence patterns - from distinct peaks in the first two years to sporadic cases in the later years. Thus, in the case of H5 in chickens, inclusion of more data for model fitting decreased prediction accuracy due to a shift in the dynamics of the system. Excluding the very early data (the sliding window approach of step-by-step B) improved out-of-sample prediction accuracy because the earlier dynamics have less weight in the prediction. In the case of H9, where there was no apparent shift in prevalence regime, the penalty for including earlier data was much less severe, although it did exist. This suggests that in dynamical systems with complex ecology such as AIV in poultry, it can be a good strategy to update model parameters with the most recent data (and exclude earlier data) if the primary objective is to forecast prevalence. However, this would only be the case in systems that continually shift to different behaviors rather than those that cycle between similar behaviors.

The apparent regime shift for H5 may partly have been due to the low isolation rates in chickens relative to other hosts species (especially geese) and was not observed when data from the same time period in other Chinese provinces were summed with the Shantou data [4]. The combination of low prevalence measured over a short sampling period could create the appearance of a regime shift if sample sizes are near the threshold of detection and there were only a few instances where underlying prevalence was high enough for detection. In general, prediction with such data is challenging due to the increased influence of stochasticity from sampling effects, which could also partly explain the lack of dependence by H5 on the dynamics of other subtypes. The most reliable predictive models of AIV will be based on surveillance data that has been collected using a sampling design that considers detection thresholds of multiple subtypes in multiple host species.

The best model of H9 included prevalence data from H4, H6 and H5. Previous analyses of the data showed that H6 and H9 tend to co-infect with each other more often than with other subtypes and H6 and H5 were the only subtypes besides H9 that tended to infect quail [4]. Thus, our finding that H6 and H5 are correlated with H9 prevalence is consistent with host adaptation and co-infection patterns. Similarly, the only subtype that was correlated with H5 prevalence was H9. The association of H4 with H9 prevalence was less clear. H4 infects ducks almost exclusively and tends to co-infect with H3 and H6 when co-infections occur. Thus, H4 could act indirectly, through H6. The association of wind speed with H5 prevalence is consistent with a recent study on equine influenza (EIV) which found that high wind speeds increased the risk of EIV infection through increased airborne spread through faster and further viral dissemination in the air [4]. A similar wind-based mechanism could increase transmission of AIV since it is known to be transmitted indirectly through air [35], [36], and a data-based model of between-farm spread suggests that wind can explain 24% of transmission over short distances (up to 25 km; [26]). Wind speed could also impact transmission through its effects on relative humidity [24], although we did not find any effects of relative humidity.

It is difficult to predict the future of biological systems using past events, even when data collection is designed for predictive modeling and the data are collected with high accuracy. The fact that our models, which are based on data collected primarily to obtain viral isolates, captured future AIV prevalence as well as they did shows that a simple statistical framework could serve as a tool for AIV control policy decisions. Moreover, from a management perspective, it is relevant to consider the qualitative fit which is remarkably good: the models captured the timing of major peaks, and did not predict outbreaks that did not occur, which is a key aspect for prevention. For example, although the model of H9 predicts a double peak at months 23 and 26, with relatively high prevalence in between, our model predicts a rise beginning at month 21 with a single peak at month 24. From a management perspective, capturing the second peak in the double-peak sequence is not important since high surveillance and interventions could be initiated at the outset of the predicted rise, which would allow preparedness for the second peak that was not captured. Similarly, the model of H5 captured the timing of major peaks and although it did predict single cases when none occurred (i.e., Figure 4, months 17 and 31), it did not predict large numbers of positive birds when they did not occur. Accuracy of the models would likely be even better if future surveillance data were collected specifically for the task of forecasting AIV prevalence. Although the surveillance data we used is the longest, most comprehensive data set of AIV in poultry, these data were collected with the goal of isolating AIVs for sequence analysis. Because sampling was non-random and biased towards locations/individuals with higher suspected risk of infection, prevalence in these data may be overestimated. This spatially non-random sampling of host species could partly explain the lack of influence of alternate host species in the prevalence patterns of H5 and H9.

In our data, the most frequent, consistent sampling interval was one month. Very few covariates showed any significant signal when lagged by one month, which is not completely surprising since the infectious period is much shorter (∼ 1 week). We would expect that potential effects from other subtypes or weather would occur on the time scale of the infectious period since this is also the maximum length of time that individuals remain in the market. We could not model lagged effects at these biologically relevant time scales (i.e., 1 week) because the sampling frequency was not high enough. Instead, we considered covariates from the same time step as the response variable since any potential lagged effects would be subsumed into the same time step. Thus, the models we presented could not be used for forecasting, *per se,* since they include covariates from the same time step as the data to be predicted.

Nevertheless, we showed that covariates selected using past data can predict future data, highlighting that a simple statistical framework could be used for predicting prevalence patterns of specific subtypes despite the complex ecological context. In order to develop statistical forecasting tools that can be applied towards anticipating the timing of outbreaks, the time interval should approximate the infectious period of the virus (weekly at most) and turnover times of different poultry types. It may be that the same number of samples with much better temporal coverage would permit a statistical model similar to those presented here but with much higher utility.

Another crucial data gap that existed in the AIV surveillance program from which we obtained our data is concurrent information on the population dynamics of each host species (i.e., rates of influx and outflow from poultry holdings). These data should be relatively easy to collect (although are subject to privacy laws in some areas) and are crucial for extending our strictly statistical method to incorporate mechanistic details such as transmission between hosts. Semi-mechanistic time series models (TSIR) have been very successful at forecasting the behavior of disease systems accurately [37], [38], [39] and wholly mechanistic models can serve as key tools for evaluating the efficacy of interventions and improving our understanding of how such perturbations may change prevalence patterns [40], [41], [42], [43]. The host species population data are especially important for understanding AIV dynamics in poultry holdings due to the rapid host turnover. For example, if we consider a farm-to-market system, the rate at which susceptible and infected hosts flow in and out of markets strongly affects the likelihood of transmission within markets because the flow rates are often faster than infectious periods (which could partly explain why we did not find the H5/H9 duck or quail prevalence data to be major drivers of H5/H9 prevalence in chickens). Thus, the prevalence of AIVs in poultry holdings that supply retail markets is an equally important data gap to fill. Other important data that should be incorporated into predictive mechanistic models in order for them to be validated prior to usage is information on vaccination programs in the different poultry species and cleaning routines in the markets. These are two factors that are likely to be strong drivers of prevalence patterns. Mechanistic models will be most useful when they are structured according to the movement patterns between poultry holdings, incorporate changes in host species composition over time and can be validated with appropriate intervention-routine and prevalence data.

Our study has shown that reasonable forecasts can be made with a statistical model based solely on historical patterns. The limitation of this approach is that it is unclear if our models will maintain reasonable accuracy in the long-term, especially if large perturbations due to weather or human intervention cause a dramatic shift in AIV dynamics. Thus, to use our approach in the long-term it may be important to periodically repeat the model selection routine in case predictor variables change. It will also be useful to collect surveillance data that would enable the development of mechanistic models that could be used to evaluate how interventions may affect prevalence and predictors of prevalence. A better mechanistic understanding of AIV prevalence in source populations and transmission within markets will help with developing models that produce reliable forecasts year after year.
